# Habitat Suitability Evaluation of Chinese Red Panda in Daxiangling and Xiaoxiangling Mountains

**DOI:** 10.3390/biology14080961

**Published:** 2025-07-31

**Authors:** Jianwei Li, Wei Luo, Haipeng Zheng, Wenjing Li, Xi Yang, Ke He, Hong Zhou

**Affiliations:** 1 Liziping Giant Panda’s Ecology and Conservation Observation and Research Station of Sichuan Province, Nanchong 637009, China; lijianwei1620@163.com (J.L.); pandaluowei@aliyun.com (W.L.); 2 Liziping Nature Reserve Administration Bureau of Sichuan Province, Ya’an 625400, China; 3 Sichuan Academy of Giant Panda, Chengdu 610081, China; zhenghp2015@163.com; 4 Sichuan Wolong National Nature Reserve Administration, A’ba 623006, China; wl-chenmeng@163.com; 5 Daxiangling Nature Reserve Management Bureau, Ya’an 625200, China; 15882601630@163.com; 6 College of Giant Panda, China West Normal University, Nanchong 637009, China

**Keywords:** *Ailurus styani*, Daxiangling mountains, Xiaoxiangling mountains, MaxEnt model, suitable habitat

## Abstract

The Chinese red panda (*Ailurus styani*) is a rare and endangered animal in China; the increase in global temperature and the interference of human activities have caused irreversible effects on the suitable habitat of wild red pandas and threatened their survival. Therefore, it is necessary to carry out scientific research and protection for Chinese red pandas. In this study, the MaxEnt model was used to predict and analyze the suitable habitats of Chinese red pandas in the large and small Xiangling Mountains. The results showed that the main ecological factors affecting the suitable habitat distribution of Chinese red pandas in the Daxiangling Mountains are the average slope (45.6%, slope), the distance from the main road (24.2%, road), and the average temperature in the coldest quarter (11%, bio11). The main ecological factors affecting the suitable habitat distribution of Chinese red pandas in the Xiaoxiangling Mountains are bamboo distribution (67.4%, bamboo), annual temperature range (20.7%, bio7), and the average intensity of human activities (8.7%, Human Footprint). The predicted suitable habitat area of the Daxiangling Mountains is 123.835 km^2^, and the predicted suitable habitat area of the Xiaoxiangling Mountains is 341.873 km^2^. The predicted suitable habitat area of the Daxiangling Mountains accounts for 43.45% of the total mountain area, and the predicted suitable habitat area of the Xiaoxiangling Mountains accounts for 71.38%. The suitable habitat area of the Xiaoxiangling Mountains is nearly three times that of the Daxiangling Mountains, and the proportion of suitable habitat area of the Xiaoxiangling Mountains is much higher than that of the Daxiangling Mountains. The suitable habitat of Chinese red pandas in the Daxiangling Mountains is mainly distributed in the southeast, and the habitat is coherent but fragmented. The suitable habitat of Chinese red panda in Xiaoxiangling Mountains is mainly distributed in the east, and the habitat is more coherent. The results of this study can provide a scientific basis for the protection of the population and habitat of Chinese red pandas in Sichuan.

## Simple Summary

The Chinese red panda is a rare and endangered species in China. The global rise in temperature and human activities have caused irreversible impacts on its habitat, making scientific research and conservation efforts essential. This study used the MaxEnt model to predict the suitable habitats of the Chinese red panda in the Daxiangling and Xiaoxiangling mountain ranges. The results indicate that the primary ecological factors affecting the Daxiangling mountain range are average slope, distance from major roads, and average temperature during the coldest quarter. For the Xiaoxiangling mountain range, the main factors are bamboo forest distribution, annual temperature variation range, and the average intensity of human activities. The predicted suitable habitat area of 123.835 km^2^ in the Daxiangling mountain range accounts for 43.45% of the mountain range’s total area, mainly distributed in the southeastern part, which is continuous but fragmented. The predicted area of 341.873 km^2^ in the Xiaoxiangling mountain range accounts for 71.38%, mainly distributed in the eastern part, which is relatively continuous. The findings provide a scientific basis for the conservation of the Chinese red panda population and its habitat in Sichuan.

## 1. Introduction

Habitat plays a crucial role in sustaining the fundamental life activities of wildlife [1,2]. In recent decades, human-induced habitat loss and fragmentation have been the primary drivers of wildlife population declines and global biodiversity loss [3,4,5,6], including factors such as overexploitation, agricultural expansion, and urbanization. Therefore, protecting suitable habitats for species is the most effective approach to conserving populations [7]. Understanding a species’ dependence on ecological conditions and spatial distribution, delineating the boundaries and characteristics of its living range, and determining how ecological factors influence its survival adaptability are key to investigating population dynamics and habitat conservation.

The red panda is a species unique to the Himalayan–Hengduan Mountains [8]. Recent evidence from population genomics has confirmed that they are two distinct species: the Himalayan red panda (*Ailurus fulgens*) and the Chinese red panda (*Ailurus styani*) [9]. They inhabit the Himalayas and its surrounding regions, including Nepal, India, Bhutan, Sikkim, Myanmar, and the southwestern provinces of China, such as Sichuan, Yunnan, and Xizang. Similar to the giant panda (*Ailuropoda melanoleuca*), the red panda is highly specialized and mainly feeds on bamboo [10]. The red panda is listed as Endangered by the International Union for Conservation of Nature (IUCN), facing greater extinction risks than the giant panda, whose status was recently downgraded from “Endangered” to “Vulnerable” [11]. However, compared to the world-renowned giant panda, the Chinese red panda has received significantly less attention from both local communities and the scientific community [12,13,14].

By the end of the last century, it was estimated that the red panda population may have declined by 40% during the latter half of the 20th century due to extensive habitat loss, increased human activities, and poaching [15]. The current population in China is estimated at 6000–7000 individuals, with approximately 3000–3400 in Sichuan [8]. In Sichuan, their distribution is mainly concentrated across six major mountain ranges [8,14]. However, according to the Fourth National Giant Panda Survey (2011–2013), red pandas were only recorded in the Qionglai, Liangshan, Daxiangling, Xiaoxiangling, and Minshan mountain ranges. The survey results also indicated that, except for a possible small remnant population in the southernmost part of the Minshan range (e.g., Mianzhu and Shifang), red pandas may have become extinct in other areas of the Minshan range (such as Pingwu and Qingchuan) [16].

The conservation crisis facing wild red pandas has drawn significant attention in China. In 1988, China implemented the Wildlife Protection Law, listing the red panda as a National Class II Protected Animal. According to incomplete statistics, by 2020, China had established 50 nature reserves within red panda distribution areas, including 37 in Sichuan, 7 in Yunnan, and 6 in Tibet. Increasing research attention has been focused on various aspects of red pandas, including their activity patterns, habitat selection, foraging strategies, and habitat quality [17,18,19,20,21]. Among these, habitat quality assessment serves as both a crucial tool for evaluating red panda survival status and an essential basis for developing habitat conservation and management strategies, making it a high-priority research area.

The conservation of the red panda has garnered attention from governments worldwide. Despite protection under international conventions and Nepal’s National Law, its population has been declining for three decades [10]. Human impacts on their habitats have been identified as the primary threat to the species’ current distribution [22,23,24,25]. Large herds of cattle, livestock farmers, and their dogs also disrupt red pandas and their habitats [26]. Panti et al.’s study on Nepal’s red pandas revealed that road distance [27], livestock density, population density, and annual average temperature are the most critical factors determining habitat suitability. In response, Nepal [28] and Bhutan [29] have launched five-year conservation action plans for red pandas [13]. However, China, India, and Myanmar have yet to establish dedicated conservation programs focused on red pandas.

The Maximum Entropy Model (MaxEnt), as a crucial tool for assessing wildlife habitat quality, is widely employed by researchers in habitat evaluation due to its high accuracy and stability [13,30]. Current habitat quality assessments for red pandas primarily focus on obtaining habitat status information at the landscape scale, aiming to understand their distribution patterns and fragmentation levels, thereby proposing targeted conservation management strategies [31,32,33]. This study utilizes the MaxEnt model to evaluate habitat suitability for red panda populations in the Daxiangling and Xiaoxiangling Mountain Ranges, while elucidating the varying degrees of influence exerted by different environmental factors on their suitable habitats. The findings aim to provide a scientific and effective theoretical foundation for the conservation and management of Chinese red panda.

## 2. Materials and Methods

### 2.1. Study Area

The Daxiangling Mountain Range extends along the southwestern edge of the Sichuan Basin, primarily composed of mid-elevation mountains with abundant rainfall and a humid climate. The average annual precipitation ranges from 1300 to 2000 mm, with temperatures consistently above 16 °C. Vegetation coverage in the area exhibits distinct altitudinal zonation as follows: below 1500 m: evergreen broad-leaved forests; 1500–2500 m: mixed coniferous and broad-leaved forests; 2500–3200 m: coniferous forests; and above 3200 m: alpine shrubs and meadows. This mountain range provides a habitat for numerous rare wildlife species, including the giant panda, Sichuan takin (*Budorcas taxicolor*), forest musk deer (*Moschus berezovskii*), Asian black bear (*Ursus thibetanus*), and Chinese red panda (*Ailurus styani*). It also harbors endangered plant species such as the dove tree (*Davidia involucrata*) [34].

The Xiaoxiangling Mountain Range is situated west of the Daliang Mountains, characterized primarily by mid-to-high mountain terrain with some low mountains and river valley terraces. It features a subtropical monsoon-influenced mountain climate, with an average annual temperature of approximately 11.7–14.4 °C and annual precipitation ranging from 800 to 1250 mm. The vegetation zones follow this general altitudinal pattern: below 2000 m: xerophytic valley shrublands; 2000–2400 m: montane broadleaf forests; 2400–3200 m and up to 4000 m: montane dark coniferous forests and subalpine dark coniferous forests. The Xiaoxiangling Range boasts rich biodiversity, including rare and endangered plant species such as the dove tree (*Davidia involucrata*), katsura tree (*Cercidiphyllum japonicum*), and Chinese tetracentron (*Tetracentron sinense*), as well as rare animals like the Chinese red panda (*Ailurus styani*), forest musk deer (*Moschus berezovskii*), and Chinese monal (*Lophophorus lhuysii*) [35].

### 2.2. Study Subject

The Chinese red panda is an endemic species to the Himalayan–Hengduan Mountains [8]. Its distribution spans the Himalayan region and adjacent areas, including Nepal, India, Bhutan, Sikkim, and Myanmar, as well as three provinces in southwestern China: Sichuan, Yunnan, and Tibet. It is classified as a Category II nationally protected animal in China. In Sichuan Province, the species is currently only found in the Qionglai, Liangshan, Daxiangling, and Xiaoxiangling mountain ranges.

### 2.3. Data Processing

#### 2.3.1. Species Data

**Figure 1:**
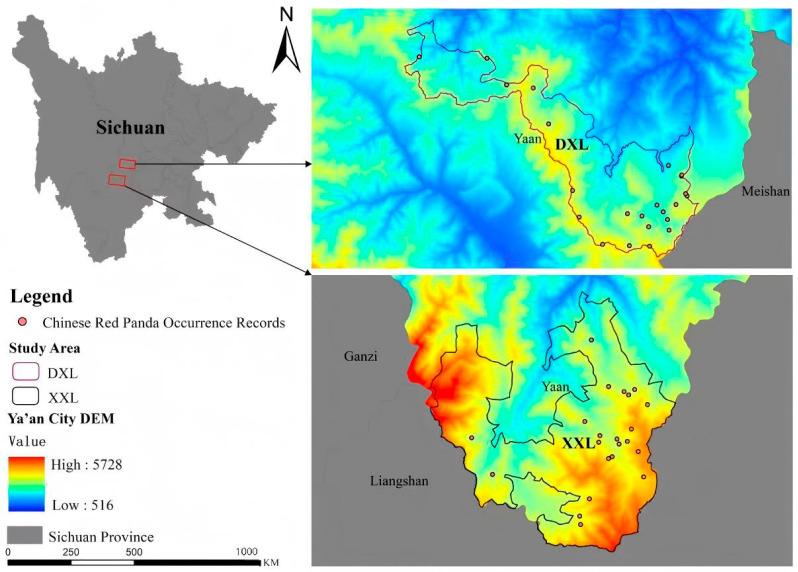
Study area and distribution sites of Chinese red pandas.

The distribution data of Chinese red pandas were obtained as follows: infrared camera monitoring at Liziping National Nature Reserve, Shimian County (July 2019–April 2023), with 22 camera sites recording red panda presence, and infrared camera monitoring at Daxiangling Provincial Nature Reserve, Yingjing County (October 2019–November 2020), with 38 camera sites recording red panda presence (Figure 1). Following Kramer-Schadt et al.’s methodology, we applied spatial filtering of occurrence records to minimize sampling bias and improve the predictive accuracy of our species distribution models. Specifically, we created 30 m × 30 m grids, and excluded duplicate records within each grid cell. This process filtered the occurrence records to 22 valid points in Liziping and 23 in Daxiangling Reserve. These spatially filtered datasets provide accurate and sufficient data for evaluating the habitat suitability of Chinese red pandas in the study area.

#### 2.3.2. Environmental Variables

This study selected four categories of environmental variables: topographic, climatic, disturbance, and vegetation factors (Table 1). Bioclimatic data are frequently chosen as variables for species distribution modeling [12,36]. Nineteen bioclimatic raster layers with a spatial resolution of 30′ (~1 km) were obtained from WorldClim version 2 (http://worldclim.org, accessed on 19 April 2024) [37]. The dataset provides monthly climate data (1970–2000) including mean minimum, mean, and maximum temperatures, as well as precipitation information [38].

Land use data were sourced from CASEarth (http://data.casearth.cn, http://worldclim.org, accessed on 17 April 2024). For analytical purposes, the land use data were classified into eight categories: coniferous forest, broadleaf forest, shrubland, grassland, wetland, cropland, residential areas, and bare land. The 2020 land use data were selected as they closely align with the temporal coverage of the infrared camera trap records of Chinese red pandas.

Topographic factors—including elevation, slope, and aspect—precisely characterize the environmental conditions of species habitats and are commonly used in habitat suitability assessments for medium- to large-sized wildlife. Topographic data were acquired from the Geospatial Data Cloud website (http://www.gscloud.cn, http://worldclim.org, accessed on 17 April 2024). A 30 m × 30 m digital elevation model (DEM) was downloaded, and slope and aspect were derived using spatial analysis tools in ArcGIS 10.8. Water resource data were generated through hydrological analysis of the regional DEM. Euclidean distance raster data were processed using the Spatial Analyst → Distance → Euclidean Distance tool in ArcGIS 10.8.

The Human Footprint dataset was downloaded from Figshare (https://figshare.com, http://worldclim.org, accessed on 17 April 2024) to assess the impact of human activities on the distribution of red panda habitats. The Human Footprint Index was derived from eight human pressure variables (built environment, population density, nighttime lights, croplands, pasturelands, roads, railways, and navigable waterways), providing a comprehensive reflection of human activity intensity [39]. Based on the Human Footprint Index values, human activity intensity was classified into four categories: no pressure (≤5), low pressure (5–10), moderate pressure (10–20), and high pressure (≥20). Changes in the Human Footprint Index were also categorized into three types: decrease (−5 to 0), increase (0–5), and significant increase (≥5).

Environmental factor data were imported into SPSS Statistics 20.0 software, and Pearson correlation coefficients were used to screen variables across different periods. When two variables were highly correlated (≥0.90), the one with greater ecological relevance was selected for subsequent modeling to reduce multicollinearity among environmental variables. Ultimately, eight climatic and environmental factors were retained for each reserve: Liziping Reserve: Bio1, Bio2, Bio7, Bio11, Bio13, Bio15, Bio18, and Bio19; Daxiangling Reserve: Bio2, Bio4, Bio7, Bio9, Bio11, Bio13, Bio15, and Bio17. All environmental factor data were standardized to the WGS_1984_UTM_Zone_48N coordinate system. Using ArcGIS, the data were masked and reclassified into 30 m × 30 m raster grids, then exported in ASCII format for subsequent MaxEnt model construction.

### 2.4. Construction and Analysis of the MaxEnt Model

The Maximum Entropy Model (MaxEnt) was employed to assess habitat suitability based on the occurrence points of red pandas and environmental variables. Among these, 75% of the data were used for training, while the remaining 25% served as test data. MaxEnt was selected because it can model species distribution using presence-only data and demonstrates superior performance compared to other species distribution models (SDMs), such as GLM, GBM, and GAM [40]. The Area Under the Curve (AUC) of the Receiver Operating Characteristic (ROC) curve was chosen to evaluate model performance. Training and test AUC values above 0.75 indicate reasonable to high discriminatory power and good model performance [41]. The Jackknife method was applied to examine the relative importance of environmental variables in determining the distribution of suitable red panda habitats within the study area. The mean of 10 computational results was analyzed to derive the Habitat Suitability Index (HSI) for red pandas. Subsequently, the potential habitats of red pandas were classified into suitable and unsuitable areas by selecting the maximum Youden Index as the threshold [42].

## 3. Results

### 3.1. Model Construction

#### 3.1.1. Results of Habitat Suitability Model Construction for Red Pandas

**Figure 2:**
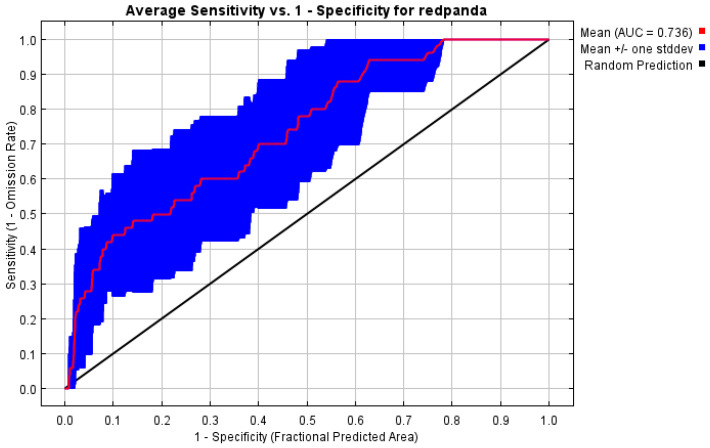
ROC verification curve of habitat suitability model of Chinese red panda in Daxiangling Mountains.

The ROC curve analysis of the MaxEnt model for red panda habitats in the Daxiangling Mountain Range showed that the AUC values for the training and test datasets reached 0.841 and 0.736, respectively. This indicates that the training model exhibits good accuracy, while the test model’s accuracy is at a moderate level. Overall, the reliability of the prediction results is high, meeting the requirements for predicting potential red panda habitats in the region (Figure 2).

**Figure 3:**
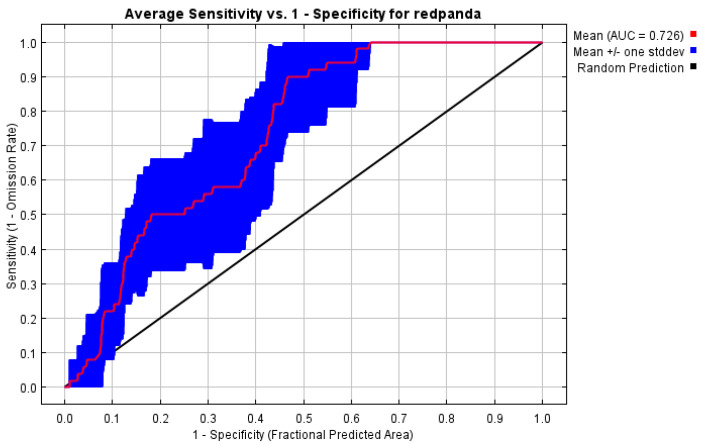
ROC verification curve of habitat suitability model of Chinese red panda in Xiaoxiangling Mountains.

The ROC curve analysis of the MaxEnt model for red panda habitats in the Xiaoxiangling Mountain Range revealed that the AUC values for the training and test datasets reached 0.814 and 0.726, respectively. This indicates that the training model demonstrates good accuracy, while the test model’s accuracy is at a moderate level. Overall, the prediction results exhibit high reliability, meeting the requirements for assessing potential red panda habitats in this region (Figure 3).

#### 3.1.2. Habitat Selection Results of Red Pandas

**Figure 4:**
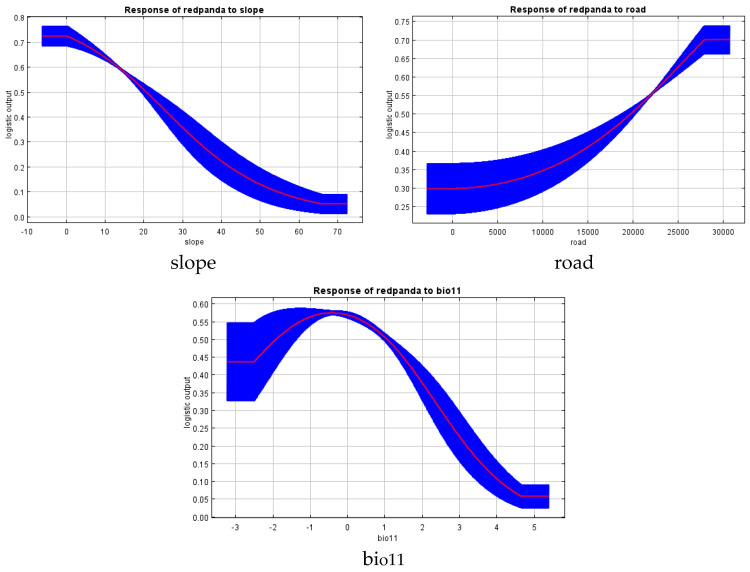
Response curve of environmental factors of Chinese red panda in Daxiangling Mountains.

**Figure 5:**
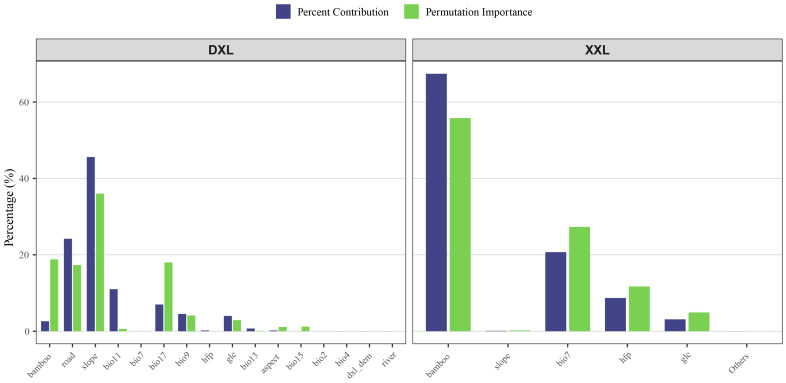
Comparison of contribution rate and arrangement importance of environmental factors.

Using the Jackknife test method integrated into the MaxEnt model, the effects of 16 environmental factors on the selection of suitable habitats for red pandas in the Daxiangling Mountain Range were analyzed. The three most influential environmental factors were average slope (45.6%, slope), distance to main roads (24.2%, road), and mean temperature of the coldest quarter (11%, bio11). The results indicate that red pandas in the Daxiangling Mountain Range tend to prefer areas with an average slope of 0–10°, located more than 27,500 m away from main roads, and with a mean temperature of the coldest quarter ranging from −2 to 1 °C (Figure 4 and Figure 5).

**Figure 6:**
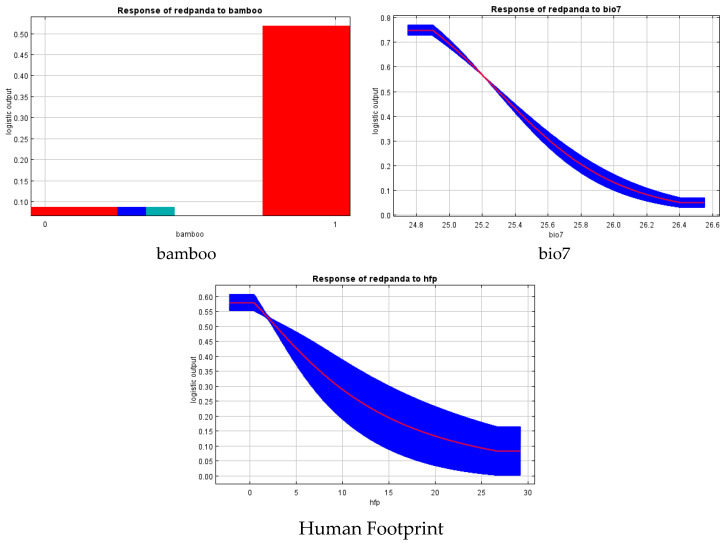
Response curve of environmental factors of Chinese red panda in Xiaoxiangling Mountains.

Using the built-in Jackknife test method of the MaxEnt model, we analyzed the effects of 16 environmental factors on suitable habitat selection for red pandas in the Xiaoxiangling Mountain Range. The results revealed three primary influencing factors: bamboo distribution (67.4%, bamboo), annual temperature range (20.7%, bio7), and mean human activity intensity (8.7%, Human Footprint). Red pandas in the Xiaoxiangling Mountain Range show a strong preference for habitats with the presence of bamboo forests, an annual temperature range around 25 °C, and low human disturbance intensity (Figure 5 and Figure 6).

### 3.2. Comparison of Suitable Habitat Areas for Red Pandas Between Daxiangling and Xiaoxiangling Mountain Ranges

**Figure 7:**
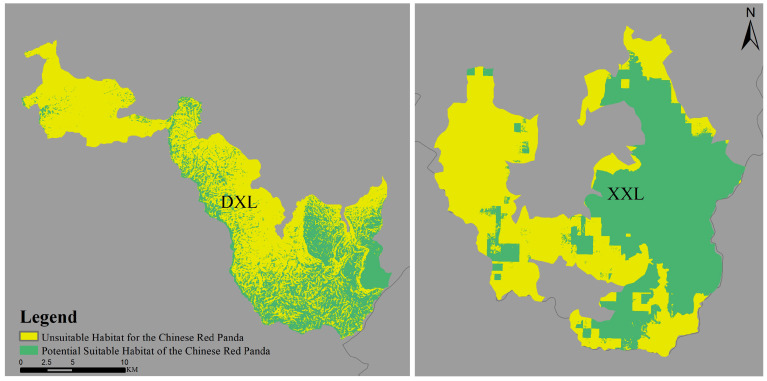
Comparison of suitable habitats for Chinese red pandas in Daxiangling and Xiaoxiangling Mountain Ranges.

After reclassifying the prediction results, the number of suitable habitat grid cells for red pandas in both mountain ranges was obtained. By multiplying the grid cell count with the individual pixel size (30 m × 30 m), the suitable habitat areas for each mountain range were calculated (Table 2). The suitable habitat area in the Daxiangling Mountain Range was 123.835 km^2^, accounting for 43.45% of its total area. In contrast, the Xiaoxiangling Mountain Range had a suitable habitat area of 341.873 km^2^, representing 71.38% of its total area. The results demonstrate that the Xiaoxiangling Mountain Range possesses a larger suitable habitat area than the Daxiangling Mountain Range, nearly three times as large; and the proportion of suitable habitat area in Xiaoxiangling is significantly higher than that in Daxiangling (Figure 7).

## 4. Discussion

Habitat serves as a crucial ecological factor in maintaining biodiversity and facilitating species evolution, with habitat suitability directly influencing species survival and reproduction [43]. Investigating the distribution of suitable habitats and their determining factors represents an important approach to understanding species’ living conditions. While numerous studies have predicted habitat suitability for red pandas (*Ailurus styani*) across different regions of Sichuan, research focusing specifically on the Daxiangling and Xiaoxiangling Mountain Ranges remains limited. In recent years, an increasing number of scholars have employed species distribution models to study wildlife habitats. The Maximum Entropy (MaxEnt) model has been widely recognized for its superior performance in predicting species distributions [44,45]. This study utilized the most recent infrared camera data from nature reserves to obtain occurrence records of red pandas in both mountain ranges, enabling accurate habitat prediction and evaluation. The test dataset AUC values of 0.736 and 0.726 demonstrated reliable model performance. The resulting habitat suitability predictions provide direct guidance for developing practical conservation strategies.

This study’s MaxEnt modeling predicted 123.835 km^2^ of suitable habitat for red pandas in the Daxiangling Mountain Range, representing 43.45% of the total area. These habitats are predominantly located in the southeastern part of the range, showing connectivity but with fragmentation. The most influential habitat selection factors were slope gradient (45.6%), distance to main roads (24.2%), and mean temperature of coldest quarter (11%). Red pandas exhibited preference for gentle slopes, remote areas far from main roads (>27,500 m), and winter temperatures between −2 to 1 °C, with natural environmental factors playing a dominant role in habitat selection. In contrast, the Xiaoxiangling Mountain Range contained 341.873 km^2^ of predicted suitable habitat (71.38% of total area), primarily distributed in the eastern sector with better connectivity. The key determinants were bamboo presence (67.4%), annual temperature range around 25 °C (20.7%), and low Human Footprint (8.7%). Notably, the suitable habitat area in Xiaoxiangling was approximately triple that of Daxiangling, with significantly higher proportional coverage. These findings differ substantially from Ruan et al.’s assessment using the 3rd and 4th National Giant Panda Survey data, which estimated suitable red panda habitats of 2701.20 km^2^ and 4056.25 km^2^ in Daxiangling and Xiaoxiangling, respectively [43]. The discrepancies likely stem from variations in study area delineation methodologies.

Climate change is expected to alter species’ geographical distribution patterns, with most species showing a tendency to migrate toward higher altitudes and latitudes [46]. Compared to human disturbance factors, red pandas exhibit lower tolerance to environmental temperature variations and greater sensitivity to climate change [43]. Both slope and climatic factors are major determinants of habitat selection for red pandas in the Daxiangling and Xiaoxiangling Mountain Ranges. Different slope gradients and climate types shape distinct vegetation compositions. As red pandas primarily feed on bamboo and prefer shrubby understories in forests, these findings align with their known habitat preferences [16].

In addition to natural environmental factors, human disturbance also exerts significant impacts on red panda habitat suitability. In the Daxiangling Mountain Range, distance to main roads ranks as the second most important factor after slope in habitat selection, while human activity intensity is the third most influential factor in the Xiaoxiangling Mountain Range. Road construction and land reclamation by humans have particularly severe effects on habitat selection, with expanded land use leading to a sharp decline in suitable habitat availability. Wei Fuwen et al. identified human activities—both direct and indirect—as the primary driver of red panda population decline [8]. Similarly, Acharya et al. and Dendup et al. concluded from habitat selection analyses across different regions that anthropogenic disturbances pose the greatest threat to red panda survival, emphasizing the need for disturbance control in conservation strategies [25,47]. Currently, climatic factors and human disturbance represent the two most critical influences on red panda habitat selection. Given that global temperature rise is an irreversible climate trend, controlling human disturbances in red panda habitats becomes even more crucial under these challenging climatic conditions.

During the analysis of suitable habitats for red pandas in the Daxiangling Mountain Range, not all recorded red panda occurrence points were located within the study area. Consequently, points outside the study region were excluded from the MaxEnt model construction. Although these external points represent actual red panda presence, the model cannot predict suitable habitat ranges beyond the defined study area. This limitation may partially explain why the predicted suitable habitat area and its proportion in the Daxiangling Mountain Range are significantly smaller than those in the Xiaoxiangling Mountain Range. Current protected area coverage fails to keep pace with shifts in species’ geographic distributions driven by global climate change and human disturbances. suggest that under present climate and land use scenarios, biodiversity hotspots are primarily concentrated within existing protected areas and their adjacent regions. Similarly, this study found that suitable habitats for red pandas in both the Daxiangling and Xiaoxiangling Mountain Ranges are predominantly located within or near protected areas.

## 5. Conclusions

Today, the establishment of nature reserves is recognized as a strategic cornerstone for biodiversity conservation [48]. Creating protected areas represents the most direct approach to mitigating human disturbances and preserving species habitats. Therefore, to effectively safeguard suitable habitats and maintain global biodiversity, it is essential to scientifically delineate protected area boundaries based on regional environmental conditions; enhance field monitoring of species activities within reserves and implement proactive interventions for future habitat conservation; and strengthen protection management in both protected zones and their surrounding areas. Our research demonstrates that Chinese red pandas inhabiting the Xiaoxiangling Mountain Range exhibit a strong preference for bamboo forests. To address this, we recommend conducting in-depth scientific studies on bamboo forest regeneration and connectivity within the region. Notably, the Daxiangling Mountain Range shows that Chinese red pandas avoid roads. Based on this finding, nature reserves should implement traffic control measures and minimize human activities along roads to prevent potential disturbances to the Chinese red panda population, and raise conservation awareness among local communities bordering protected areas.

## Figures and Tables

**Table 1:** Environmental factors for assessment of red panda habitat suitability.

Factor type	Environmental Factor	Factor Description	Unit
Climate	Bio1	Average annual temperature	°C
	Bio2	Average monthly temperature difference between day and night	°C
	Bio4	Temperature variation variance	°C
	Bio7	Annual temperature variation range	°C
	Bio9	Maximum dry quarterly average temperature	°C
	Bio11	Average temperature in the coldest quarter	°C
	Bio13	Maximum monthly rainfall	mm
	Bio15	Precipitation seasonality	mm
	Bio17	Maximum seasonal rainfall	mm
	Bio18	Average rainfall in the hottest season	mm
	Bio19	Average rainfall in the coldest season	mm
Terrain	Altitude	The average elevation of the grid cell	m
	Slope	The average slope of the grid cell	°
	Road	Distance from the main road	m
	Aspect	The average slope of the grid cell	°
Disturbance	Human Footprint	The average intensity of human activity within a grid cell	
Vegetation	Vegetation	Divided into eight categories: cropland (CL), coniferous forest (NF), broad-leaved forest (BF), shrub (SL), grassland (GL), wetland (WT), residential land (RL), and bare land (BL)	
	Bamboo	Binary variable (0—no, 1—yes)	

**Table 2:** Predicted area of suitable habitat for Chinese red panda.

Mountains	Predicted Habitat Area (km^2^)	Predicted Area Ratio (%)
DXL	123.835	43.45
XXL	341.873	71.38

## Data Availability

Data will be made available upon request.

## Abbreviations

The following abbreviations are used in this manuscript:

DXL	Daxiangling
XXL	Xiaoxiangling
